# Long-Term Stability of New Co-Amorphous Drug Binary Systems: Study of Glass Transitions as a Function of Composition and Shelf Time

**DOI:** 10.3390/molecules21121712

**Published:** 2016-12-14

**Authors:** Luz María Martínez, Marcelo Videa, Nahida González Sosa, José Héctor Ramírez, Samuel Castro

**Affiliations:** 1 School of Engineering and Sciences, Tecnologico de Monterrey, Campus Monterrey Av. Eugenio Garza Sada 2501 Sur. Monterrey N.L., México 64849, Mexico; mvidea@itesm.mx; 2 Department of Chemistry and Nanotechnology, Tecnologico de Monterrey, Campus Monterrey Av. Eugenio Garza Sada 2501 Sur. Monterrey N.L., México 64849, Mexico; nahidaglz@gmail.com (N.G.S.); jose.ramirez.hs@gmail.com (J.H.R.); samuel.castrop@itesm.mx (S.C.)

**Keywords:** DSC, phase-diagrams, *T*_g_, co-amorphous drugs, thermal stability, nimesulide, nifedipine, carvedilol, cimetidine, structural relaxation

## Abstract

The amorphous state is of particular interest in the pharmaceutical industry due to the higher solubility that amorphous active pharmaceutical ingredients show compared to their respective crystalline forms. Due to their thermodynamic instability, drugs in the amorphous state tend to recrystallize; in order to avoid crystallization, it has been a common strategy to add a second component to hinder the crystalline state and form a thermally stable co-amorphous system, that is to say, an amorphous binary system which retains its amorphous structure. The second component can be a small molecule excipient (such as a sugar or an aminoacid) or a second drug, with the advantage that a second active pharmaceutical ingredient could be used for complementary or combined therapeutic purposes. In most cases, the compositions studied are limited to 1:1, 2:1 and 1:2 molar ratios, leaving a gap of information about phase transitions and stability on the amorphous state in a wider range of compositions. In the present work, a study of novel co–amorphous formulations in which the selection of the active pharmaceutical ingredients was made according to the therapeutic effect is presented. Resistance against crystallization and behavior of glass transition temperature ($T_{g}$ were studied through calorimetric measurements as a function of composition and shelf time. It was found that binary formulations with $T_{g}$ temperatures higher than those of pure components presented long-term thermal stability. In addition, significant increments of $T_{g}$ values, of as much as 15 ${}^{\circ }$C, were detected as a result of glass relaxation at room temperature during storage time; this behavior of glass transition has not been previously reported for co-amorphous drugs. Based on these results, it can be concluded that monitoring behavior of $T_{g}$ and relaxation processes during the first weeks of storage leads to a more objective evaluation of the thermomechanical stability of an amorphous formulation.

## 1. Introduction

It is well-known that the number of active pharmaceutical ingredients (APIs) with high therapeutic potential but low water solubility is constantly growing due to sustained drug discovery efforts. Currently, around 40% of commercial drugs are sparingly soluble in water. The disadvantage of formulating drugs with poorly soluble active ingredients is that they can lead to low bioavailability [1]. Transformation of a material from the crystalline state into its amorphous state is a strategy applied to increase the solubility of pharmaceutical products; glassy or amorphous materials are therefore of great interest in the pharmaceutical field particularly because their preparation by melt-quenching has advantages of shorter processing time compared to other strategies [2,3,4]. The structure of an amorphous material is characterized by a long range disordered arrangement of its molecules leading to a higher chemical potential compared to the more stable crystalline form. This higher chemical potential is the driving force for a higher dissolution rate and saturation concentration when dissolved in water [5,6], but it is also the driving force for the crystallization process. Due to their thermodynamic instability, active pharmaceutical ingredients in the amorphous state tend to recrystallize [7,8,9]. In order to avoid crystallization, it has been a common strategy to add a second component to hinder the crystalline state and form a thermally stable co-amorphous system, that is to say, an amorphous binary system which retains its amorphous structure. The second component can be a small molecule excipient (such as a sugar or an amino acid) or a second drug, with the advantage that a second component could be used for complementary or combined therapeutic purposes [2]. A review of the current literature shows that efforts along this line are modest, there being around twenty drug–drug binary systems reported to be stable in the amorphous state that have been studied thermally and structurally [3,10]. If we compare this number with the large number of poorly soluble drugs [11], there is a great area of opportunity to find new stable amorphous binary systems with increased solubility. In addition, most of the studies on co-amorphous drugs report limited compositions of the binary systems, at most 1:1, 1:2 and 2:1 ratios, leaving a gap of information that needs to be filled by the exploration of a wider composition range to fully characterize the effects of the formulation on the stability and phase transitions of the mixtures, both in the crystalline and amorphous state.

The reason why most of the studies of binary systems are performed in the 1:1 molar ratio is that this composition has been considered the most stable in the amorphous mixture; in some cases, it has been reported that an excess of either API in the sample may destabilize the system enough as to return it to its crystalline state. The hypothesis for a preferable 1:1 formulation is based on the idea that heterodimers are formed through intermolecular interactions, like hydrogen bonding or ion pairing, leading to a structure that is unable to find a new crystalline order [12,13].

The lack of systematic studies reporting long-term thermal stability of binary systems against crystallization in a wide range of compositions has motivated us to establish a strategy for the selection of the components of a binary system based on the construction of phase diagrams and the behavior of glass transition temperatures as a function of composition. With this knowledge, it is possible to define the feasibility of stable formulations of a binary system of active ingredients at a ratio relevant to complementary therapeutic doses.

The aim of this study was to prepare and evaluate the thermal properties of new co-amorphous binary systems whose components (nifedipine, NIF; nimesulide, NIM; carvedilol, CAR and cimetidine, CIM) were selected considering potential pharmaceutical interest in formulations of these APIs for combined or complementary therapy. Cimetidine was chosen because it is one of the most prescribed drugs for treatment of gastric ulcers and reflux diseases and lately has been reported as a possible anticancer agent [14]. In order to take advantage of two drugs in a co-amorphous system, and since there is a considerably high number of patients with cimetidine treatment, there is an obvious need to study this API in combination with other drugs. In the present work, cimetidine was studied as a second component of a binary system with nifedipine, a class II drug (low solubility and high permeability) that is unstable in the amorphous state. Nifedipine is used for the treatment of angina pectoris and primary hypertension. There are reports that cimetidine causes increments of pharmacokinetics of nifedipine with no apparent effect on the pharmacological response [15]; keeping this information in mind, it is of interest to explore the stability of these two APIs in a wide range of compositions in order to find possible combined formulations.

In relation to NIM-CAR, nimesulide is also a class II drug, and one of the most commonly prescribed drugs for its anti-inflammatory , antipyretic, and analgesic activities [16]. It belongs to a sulfonanilide compound class with a low incidence of side effects. It is indicated for patients with disorders such as arthritic conditions, musculoskeletal disorders, headache and vascular diseases [17]. Carvedilol is a class II drug used as an antihypertensive agent and in the treatment of heart failure [18]; considering that there is a high number of patients that may present both illnesses, carvedilol was selected to be studied in combination with nimesulide.

Phase transitions of the new binary systems were fully characterized on a wide range of molar ratios. Preparation and characterization of the new co-amorphous system were performed in order to study the long-term stability on the amorphous state as a function of behavior of their glass transition temperatures, composition and shelf time.

## 2. Results

As an initial step towards the characterization of the proposed systems, samples of pure APIs (NIF, NIM, CAR, and CIM) shown in Table 1 were prepared to evaluate the thermal properties of their crystalline and amorphous state. Two new binary co-amorphous systems were prepared, NIF-CIM and NIM-CAR, whose components were selected according to their therapeutic complementarity. These mixtures were prepared in a wide range of molar fractions to construct phase diagrams and to gather enough information to evaluate the effect of composition in the glass transition temperature and stability of the amorphous state. The measurements of the glass transition temperatures after a long storage time showed a significant increase in $T_{g}$ compared to the values observed right after the preparation of the glass, indicating a structural relaxation process as a result of storage time. This was monitored as a function of time with the interest of describing the effect of this relaxation in the stability of the amorphous state.

**Figure 1:**
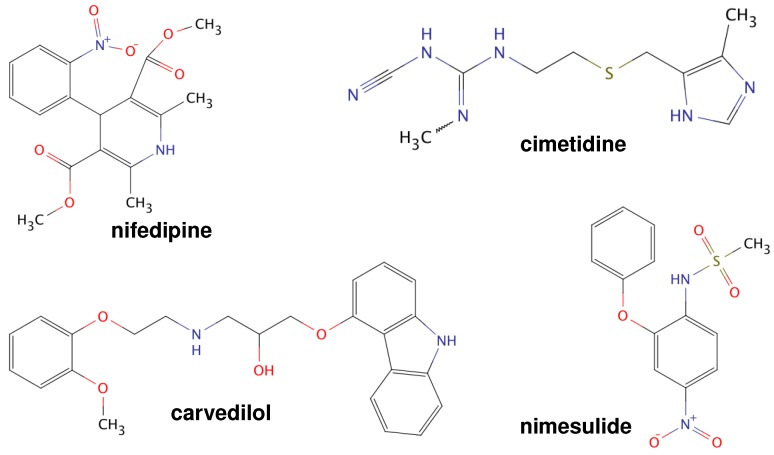
Chemical structures of carvedilol (CAR), cimetidine (CIM), nifedipine (NIF) and nimesulide (NIM).

Table 1 also shows properties of the pure active ingredients selected in this work. The chemical structures of these substances are shown in Figure 1.

### 2.1. Thermal Characterization

#### 2.1.1. The NIF-CIM System

**Figure 2:**
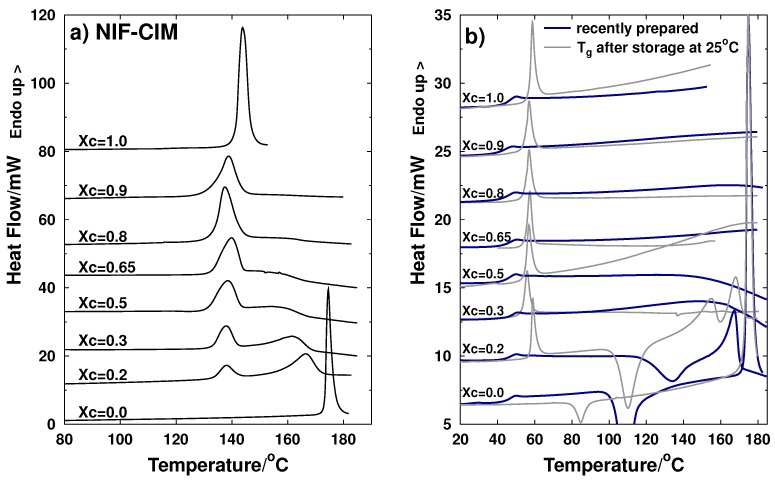
Thermograms of the NIF-CIM binary system obtained by Differential Scanning Calorimetry (DSC). The mole fractions are expressed in terms of cimetidine ($X_{c}$) (**a**) crystalline samples (**b**) amorphous samples: plots with **blue** thick lines correspond to the measurement of the glass transition temperature performed immediately after the quenching of the molten sample. Thin **gray** lines correspond to the measurements performed after 133 days of storage at 25 ${}^{\circ }$C. (except for samples $X_{c}=0.3$ and $X_{c}=0.65$ that correspond to 80 days and 40 days of storage, respectively). Heating rate was 10 ${}^{\circ }$C/min in all cases.

Figure 2a shows the thermograms corresponding to the heating process of crystalline samples of the NIF-CIM system. For the pure components, the endothermic peak corresponds to the melting of the active ingredient (mol fraction $x_{c}=1.0$ corresponds to cimetidine and mol fraction $x_{c}=0$ corresponds to nifedipine). Melting temperature of CIM occurred at 141.5 ${}^{\circ }$C, which corresponds to the forms A or D of polymorphs of this drug; these two polymorphs have very close melting temperatures according to the results reported by Bauer-Brandl et al. [19]. For pure nifedipine, the endothermic peak corresponding to its melting temperature occurred at 172 ${}^{\circ }$C; it has been reported that there are three polymorphs of this drug, and according to data reported, this peak corresponds to melting process of polymorph Form I or α [8,25,26]. In the case of crystalline mixtures, the first endothermic peak corresponds to the melting of the eutectic composition and the second to the *liquidus* temperature. The eutectic temperature or invariant temperature corresponds to the lowest temperature at which a specific proportion of the components of the binary mixture start to melt (eutectic composition). As the temperature is increased, the proportion of the liquid phase present gradually increases until the whole sample is molten at the *liquidus* temperature.

Figure 2b shows the thermograms resulting from the thermal analysis of the amorphous in situ quenched samples where the blue lines correspond to the measurements performed immediately after preparation of the amorphous samples in differential scanning calorimetry pans. According to the heating curves, no crystallization process was observed during heating for pure CIM ($x_{c}=1$), and it showed a glass transition temperature signal at 43.6 ${}^{\circ }$C. For the case of NIF ($x_{c}=0$), the heating scan performed immediately after preparing an amorphous sample also showed a $T_{g}$ signal of around 43 ${}^{\circ }$C and also an exothermic peak at 98 ${}^{\circ }$C due to crystallization while heating up. The binary system with molar fraction $x_{c}=0.2$ also showed $T_{g}$ and a crystallization process which indicates that this particular composition is also unstable when sample is heated; this can be explained by the fact that this mixture is rich in NIF. It is worth mentioning that, although the sample with composition $x_{c}=0.2$ crystallizes when heated, it was stable during storage at 25 ${}^{\circ }$C. Curves with mole fractions from $x_{c}=0.3$ to $x_{c}=0.9$ showed very similar values of $T_{g}$ and none of these systems presented a crystallization process, indicating that these amorphous binary samples are stable against crystallization. These results showed that stabilization of the amorphous state of NIF can be achieved by adding CIM (in a composition range $x_{c}=0.3$–0.9).

After storing the samples at room temperature in dry conditions (in a desiccator at 25 ${}^{\circ }$C) for 133 days, a second measurement was performed. These thermograms are shown with gray thin lines (Figure 2b). A higher glass transition temperature and an overshoot are observed for each of the stored samples, except for pure nifedipine which shows almost complete crystallization (close to 90% of the amorphous material was lost due to spontaneous crystallization). The overshoot observed during the glass transition measurement corresponds to the recovery of the lost enthalpy as a result of a spontaneous molecular reorganization of the amorphous material in which its structure shifts towards its thermodynamic equilibrium [27] .This molecular reorganization also explains the increment of about 5 to 15 degrees in the glass transition temperatures of CIM and CIM-NIF mixtures. An increment of the glass transition temperature has been reported by Pikal et al. in studies of annealing of sugar glasses performed at several temperatures [28], but a similar observation has not been previously reported for active pharmaceutical ingredients during storage at room temperature. For stored samples with compositions from $x_{c}=0.3$ to $x_{c}=1$, the absence of crystallization and melting endotherms indicate stability in the amorphous state during storage of these binary mixtures.

#### 2.1.2. NIM-CAR System

**Figure 3:**
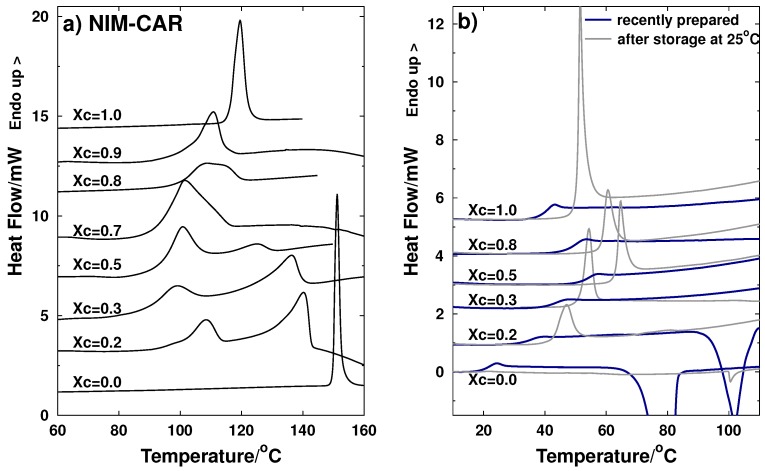
Thermograms of the NIM-CAR binary system obtained by DSC. The mole fractions are expressed in terms of carvedilol ($X_{c}$) (**a**) crystalline samples (**b**) amorphous samples: plots with **blue** lines correspond to the measurement of the glass transition temperature performed immediately after the quenching of the molten sample. Thin **gray** lines correspond to the measurements carried out after 85 days of storage (at 25 ${}^{\circ }$C), except for sample $X_{c}=0.3$ in which storage time corresponds to 39 days . Heating rate was 10 ${}^{\circ }$C/min in all cases.

Figure 3a shows the thermograms corresponding to the heating process of crystalline samples of the NIM-CAR system. In a similar manner as the NIF-CIM system, the onset of the endothermic peaks from the pure components correspond to the melting temperatures of each active ingredient. For each composition of the binary mixtures, the onset of the first endothermic process corresponds to eutectic temperature and the peak of the second endothermic process is taken as the liquidus temperature.

Figure 3b shows thermograms of amorphous samples obtained by melt-quenching. For the recently prepared samples, it can be observed that NIM shows a crystallization peak at 78 ${}^{\circ }$C, consistent with the value reported previously by Pajula et al. of 79 ${}^{\circ }$C [29]. Nimesulide is unstable in an amorphous state, and it can be observed that stabilization of the amorphous state can be achieved by adding carvedilol, since, for compositions from $x_{c}=0.3$ to $x_{c}=0.8$, only a glass transition process is observed and no crystallization process is present. Together with the thermograms for the samples measured immediately after preparation, Figure 3b also shows the thermograms of stored samples (85 days of storage at 25 ${}^{\circ }$C). As it was observed for the NIM-CIM system, the thermograms of the stored NIM-CAR samples also present a clear overshoot at the glass transition due to the structural relaxation of the material. Once again, the glass transition temperatures are in all cases higher by almost 10 ${}^{\circ }$C in the stored samples compared to the values measured right after the quenching.

#### 2.1.3. Phase Transition Diagrams

**Figure 4:**
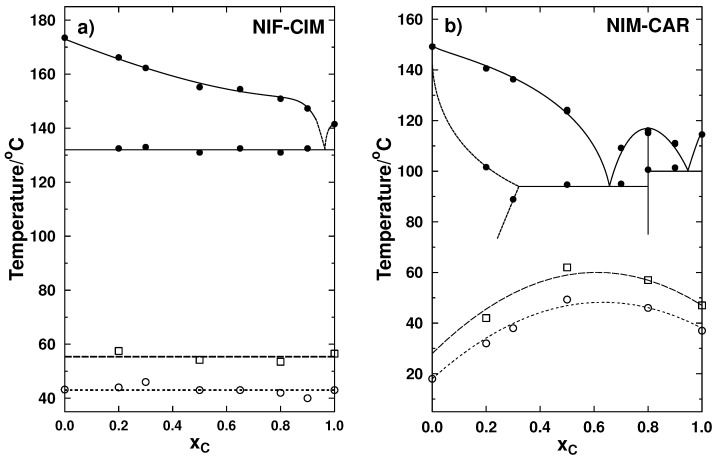
Phase transition diagrams for the binary systems. Liquidus and eutectic temperatures are shown with filled circles. In the phase diagrams are also included the glass transition temperatures measured immediately after the quenching (empty circles) and after storage (empty squares) for (**a**) NIF-CIM showing glass transition temperatures at 0 and 133 days of storage and (**b**) NIM-CAR at 0 and 85 days of storage.

From the processes observed in the thermograms for the samples in the crystalline state (see Figure 2a), a phase diagram was constructed for the system NIF-CIM, and it is presented in Figure 4a. $T_{g}$ values for samples right after prepared (just after quenching) and after 133 days of storage are also included in the same phase diagram. Glass transition temperatures for the pure components and for several of the compositions prepared are very similar and are near 43 ${}^{\circ }$C. In the case of the stored samples, the $T_{g}$ is observed at a higher temperature at values close to 56 ${}^{\circ }$C, resulting in an increment of 13 ${}^{\circ }$C. This is an important finding because, in most cases, $T_{g}$ of co-amorphous systems are evaluated only right after the amorphous material has been prepared and measurements should be made after enough time has passed as to reach a stable $T_{g}$ temperature corresponding to a relaxed glassy structure. This time may vary for each sample, but for organic molecules, which are typically classified as fragile liquids, three to four weeks may be needed. It is important to mention that, although there are observations of relaxation as a result of exposing amorphous samples to annealing processes in previous studies [30], there are no prior reports of increments in $T_{g}$ during storage at room temperature for amorphous drugs.

From the transition temperatures observed in the thermograms shown in Figure 3a, a phase diagram for NIM-CAR was constructed in which the glass transition temperatures for the recently prepared amorphous samples and those measured after a storage period of 85 days are also included. It can be observed that, in this binary system, the glass transition temperatures are higher for the mixtures than for the pure components, and, for all stored samples, an increment of $T_{g}$ was observed of about 11 ${}^{\circ }$C.

The increments in $T_{g}$ during storage at room temperature have not been reported for co-amorphous systems, since, in most cases, $T_{g}$ is only evaluated right after the amorphous material has been prepared. These findings suggest that measurements of glass transition should be made after enough time has passed as to reach a more relaxed glassy structure. To study this increment of $T_{g}$ during storage, a detailed monitoring of this phenomenon was performed.

## 3. Discussion

### 3.1. Behavior of Glass Transition during Storage

**Figure 5:**
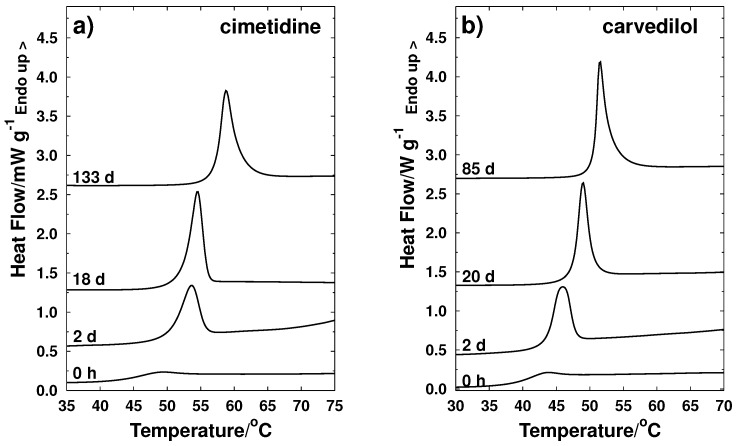
Behavior of glass transition temperature as a function of storage time (days) for amorphous pure active ingridients: (**a**) cimetidine; (**b**) carvedilol.

**Figure 6:**
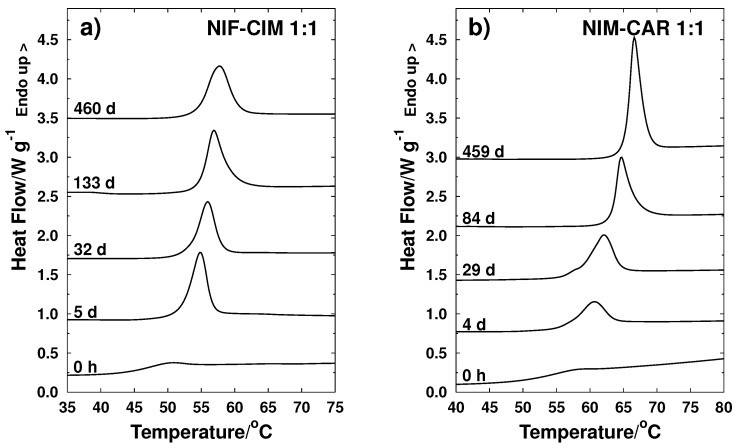
Behavior of glass transition temperature as a function of storage time (days) for amorphous binary systems: (**a**) NIF-CIM and (**b**) NIM-CAR. For both systems, the molar ratio is 1:1.

Measurements of $T_{g}$ after a certain storage time were performed to study behavior of glass transition and relaxation processes for samples stored at 25 ${}^{\circ }$C. Results are shown in Figure 5 for pure cimetidine and carvedilol (nimesulide and nifedipine samples presented crystallization and therefore are not included in this analysis) and Figure 6 for the binary mixtures NIF-CIM and NIM-CAR of composition $x_{c}=0.5$.

**Figure 7:**
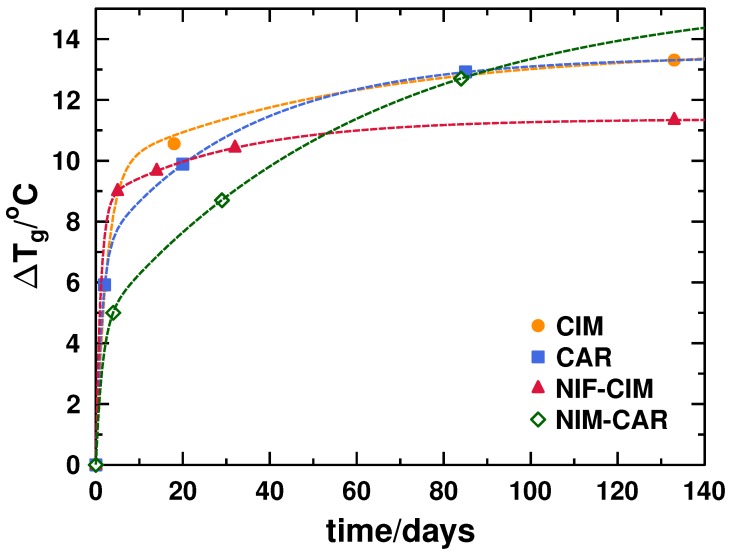
Behavior of glass transition temperature (Δ$T_{g}$ is the difference between $T_{g}$ of stored sample and $T_{g}$ of freshly prepared sample, t=0 d) as a function of storage time.

The analysis of the behavior of $T_{g}$ as a function of time, presented in Figure 7, shows a two-step relaxation process: one that occurs relatively fast, generally responsible for the largest increment of $T_{g}$, and a second step, showing a slower relaxation process whose change in $T_{g}$ is not as significant as the first step. With these results, it is concluded that measurements should be made after enough time has passed as to allow the fast relaxation process to occur. This time may vary for each sample, but it seems that, for organic molecules, which are typically classified as fragile liquids, ten to twenty days may be needed. It is important to mention that, although there are observations of relaxation as a result of exposing amorphous samples to annealing processes in previous studies [30], there are not prior reports of observations of increments in $T_{g}$ for amorphous drugs during shelf life at room temperature.

**Figure 8:**
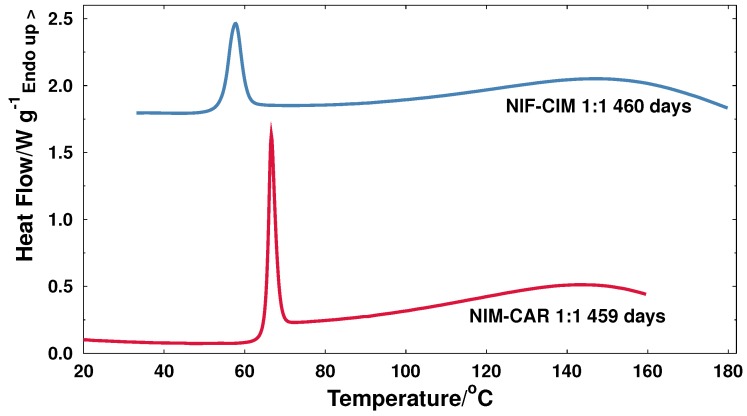
Long-term stability of binary mixtures after storage of more than 15 months at room temperature shown in the DSC thermograms.

The stability of two unstable active ingredients in amorphous state (NIM and NIF) was markedly increased to almost two years by formulating them as co-amorphous binary systems. As evidence of the long-term stability of the new co-amorphous formulations presented in this work, Figure 8 shows thermograms of binary systems that have remained stable in the amorphous state for a period longer than fifteen months (samples are still stable and still being monitored).

### 3.2. Spectroscopic Analysis

Fourier transform infrared (FTIR) spectra of pure active ingredients in crystalline and amorphous state were acquired in order to gather additional information on the structural modifications caused by loss of directional order and changes in intermolecular interaction. In addition, spectra of binary amorphous mixtures of molar composition 1:1 are presented for comparison to the spectra of pure amorphous samples.

**Figure 9:**
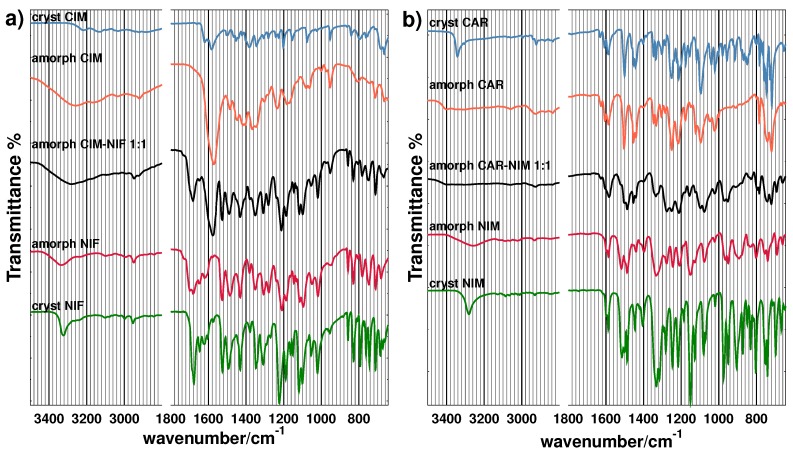
Fourier transform infrared (FTIR) spectra for crystalline and amorphous samples of pure active ingredients and amorphous 1:1 mixture for the systems (**a**) cimetidine–nifedipine and (**b**) carvedilol–nimesulide.

As it can be seen in Figure 9a, a broadening of the signal corresponding to the –NH functional group of cimetidine is observed going from the crystalline to the amorphous state with a corresponding shift from 3220 to 3260 cm${}^{-1}$. This shift to a higher wavenumber suggests a loss of hydrogen bonding between the amino hydrogen and the nitrogen in the guanidine group. This observation correlates with the merge of cimetidine signals at 1620 and 1580 cm${}^{-1}$, associated with a $sp^{2}$ C–N double bond, and merges in a strong single band in the amorphous state. Signals at 1200, 1160 and 1080 cm${}^{-1}$, attributable to the stretching of the $sp^{2}$ C–N single bond, also show a significant broadening in the amorphous state. Similarly, in the case of crystalline nifedipine, the –NH signal in the crystalline state at 3220 cm${}^{-1}$ shifts to 3340 cm${}^{-1}$, while the ester –C=O absorption occurring at 1680 cm${}^{-1}$ in the crystalline state increases to 1700 cm${}^{-1}$, indicating a loss of hydrogen bonding interactions due to molecular disorder. The symmetric –NO${}_{2}$ signal at 1310 cm${}^{-1}$ apparently splits into two signals at 1280 and 1300 cm${}^{-1}$ in the amorphous state.

After inspection of the spectrum for the amorphous CIM-NIF binary mixture, it can be deduced that, in general, all IR signals are in good approximation a superposition of signals present in amorphous cimetidine and nifedipine, except for the broad band observed in amorphous nifedipine from 1600 to 1700 cm${}^{-1}$, which is reduced to a narrower band around 1680 cm${}^{-1}$ in the mixture.

In Figure 9b, it can also be found that the combined –NH and –OH signal in carvedilol presents a shift to a higher wavenumber, from 2940 cm${}^{-1}$ to 3400 cm${}^{-1}$, suggesting a weakening in the hydrogen bonding in the amorphous state. Above 1400 cm${}^{-1},$ crystalline and amorphous carvedilol are not significantly different and signals below 1000 cm${}^{-1}$ observed in the crystal are missing in the amorphous state. Comparing crystalline and amorphous nimesulide, all signals are in general broadened and weakened. The –NH signal of the sulfonamide shifts from 3280 cm${}^{-1}$ to a broad signal at 3260 cm${}^{-1}$. In this case, the slight shift observed is consistent with an intramolecular hydrogen bond that is not greatly disturbed by the molecular disordering introduced by amorphization.

In the amorphous 1:1 mixture, an apparent shift of the signals corresponding to nimesulide at 1100 and 1120–1150 cm${}^{-1}$ of about 50 cm${}^{-1}$ is observed, probably merging into signals belonging to carvedilol present in the same range.

## 4. Materials and Methods

### 4.1. Materials

Nifedipine (98%), cimetidine, carvedilol (USP) and nimesulide were purchased from Sigma-Aldrich (St. Louis, MO, USA), and they were used as received without further purification. The co-amorphous binary systems (NIF-CIM, NIM-CAR) were prepared by melt-quenching. The chemical structures of these substances are shown in Figure 1.

### 4.2. Determination of Phase Transitions by Thermal Analysis

To identify phase transitions of pure components and mixtures in the amorphous and crystalline samples, a Diamond Perkin-Elmer Differential Scanning Calorimeter, equipped with an intra-cooler system, (Waltham, MA, USA) was used. The amount of sample for thermal analysis was ca. 3 to 5 mg, which was packed and sealed in aluminum cells with a volume of 50 μL. The instrument was calibrated with Indium.

The heating and cooling method applied during thermal analysis to identify phase transitions of crystalline samples of pure active ingredients was as follows: samples were heated at a rate of 10 ${}^{\circ }$C/min from 30 ${}^{\circ }$C until samples were completely molten. After identification of endothermic signal corresponding to melting temperature, molten samples were cooled in the DSC instrument at a cooling rate around 70 ${}^{\circ }$C/min. The purpose of this cooling step was to produce amorphous samples in situ by the method of melt-quenching. Once amorphous samples were obtained, a second heating scan at 10 ${}^{\circ }$C/min was performed to identify the glass transition temperature of the recently prepared amorphous material. For the case of the binary systems, mixtures with different composition were prepared by gently grinding solids in a mortar and then analyzed in a similar fashion as the pure components.

To monitor the behavior of glass transition temperature as function of shelf-time, amorphous samples in sealed DSC cells were stored in a desiccator at room temperature (25 ${}^{\circ }$C) and then analyzed after days of storage.

### 4.3. FTIR Spectroscopic Analysis

For the spectroscopic analysis, a Fourier transform infrared spectrometer, Perkin–Elmer Spectrum 400 FTIR-NIR operating in the near-infrared region (Shelton, CT, USA) was used. Samples were placed in contact with a horizontal attenuated total reflectance (ATR) accessory (Shelton, CT, USA) with a zinc selenide prism. All spectra were acquired using four scans with a resolution of 4 cm${}^{-1}$. The range selected was from 380 to 4000 cm${}^{-1}$. For amorphous samples, 5 mg of the crystalline sample were placed on an aluminum foil, melted in an oven and cooled at room temperature to vitrify. Amorphous samples that adhered to the aluminum foil were then immediately placed on the ATR prism for measurement.

## 5. Conclusions

Stabilization of amorphous nifidipine and nimesulide (that are unstable APIs on the amorphous state as pure ingredients) was achieved by the preparation of new co–amorphous formulations, NIF-CIM and NIM-CAR, that are potential candidates for future use as combined therapy.

It was found that $T_{g}$ of binary systems can increase as a function of storage time, so it is recommended to monitor the behavior of this parameter after storage (at least three weeks after preparation) in order to obtain a more objective value of the thermomechanical stability of the samples.

Considering that, for the amorphous state, the solubility of active ingredients is enhanced, compositions of new formulations intended for its use as therapeutic formulations will have to be adjusted. For this reason, in order to develop new co-amorphous drugs for combined therapy, the study of phase transitions in a wide range of compositions (both on the amorphous and crystalline states) as well as the monitoring of $T_{g}$ as a function of storage time is necessary to make sure the systems will remain stable at complementary therapeutic doses.

## Figures and Tables

**Table 1:** Thermal properties, therapeutic activity and long-term stability of the amorphous state of active ingredients.

Pharmaceutical	$T_{g}$/${}^{\circ }$C	Polymorph	$T_{m}$/${}^{\circ }$C	Therapeutic	Long-Term Stability	References
Ingredient				Activity	(Amorphous State)	
Cimetidine	36	Form A or D	141–143	anti-ulcer agent	not studied	[12,19]
C${}_{10}$H${}_{16}$N${}_{6}$S		Form C	83			
Nifedipine	45	Form α	172	antianginal/	unstable	[20]
C${}_{17}$H${}_{18}$N${}_{2}$O${}_{6}$		Form β	170	antihypertensive		
Carvedilol	39	Form I	123–126	antihypertensive/	stable	[21,22]
C${}_{24}$H${}_{26}$N${}_{2}$O${}_{4}$		Form II	114–115	heart failure treatment		
Nimesulide	20	Form I	144	anti-inflammatory	unstable	[23,24]
C${}_{13}$H${}_{12}$N${}_{2}$O${}_{5}$S		Form II	140			

## Abbreviations

The following abbreviations are used in this manuscript:

API: Active pharmaceutical ingredient
ATR: Attenuated total reflectance
CAR: carvedilol
CIM: cimethidine
DSC: Differential scanning calorimetry
FTIR: Fourier transform infrared spectroscopy
NIF: nifedipine
NIM: nimesulide
NIR: near infrared
USP: United States Pharmacopeia
